# Dengue research: a bibliometric analysis of worldwide and Arab publications during 1872–2015

**DOI:** 10.1186/s12985-016-0534-2

**Published:** 2016-05-06

**Authors:** Sa’ed H. Zyoud

**Affiliations:** Poison Control and Drug Information Center (PCDIC), College of Medicine and Health Sciences, An-Najah National University, Nablus, 44839 Palestine; Department of Clinical and Community Pharmacy, College of Medicine and Health Sciences, An-Najah National University, Nablus, 44839 Palestine

**Keywords:** Dengue, Bibliometric, Scopus, Arab world, Citations

## Abstract

**Background:**

Dengue is an important emerging and re-emerging arboviral infection globally as a rapidly growing and widespread public health problem, with transmission occurring in more than 128 countries in Asia, Americas, southeast Africa, western Pacific, and eastern Mediterranean regions. Therefore, the aim of this study was to characterize and quantify the scientific output of dengue research in Arab countries relative to that worldwide by using a bibliometric analysis.

**Methods:**

The standardized search approach based on the use of the the keyword “dengue” in the title, abstract, and keyword field was used to get research output related to dengue at a global level. All data related to dengue were collected from the past to December 31, 2015.

**Results:**

A total of 19,581 dengue-related documents identified in the Scopus database. The results show that the study of dengue exhibits an overall upward trend from 1872 to 2015 with peak publications in 2014. The leading countries in dengue research were the USA (4,709; 24.05 %), India (1,942; 9.92 %), Brazil (1,530; 7.81 %), Thailand (1,260; 6.43 %), the UK (1,129; 5.77 %), and France (1,087; 5.55 %). Only 226 (1.16 % of the overall global research effort in the dengue field) articles were published from the Arab region. The total number of citations for all publications was 352,710, with an average of 18.0 citations per publication. Furthermore, the *h*-index for all extracted data related to dengue research was 186. Kingdom of Saudi Arabia (KSA) was the most productive country in Arab region with 102 documents representing 45.1 %. Furthermore, the *h*-index for all extracted data related to dengue research was 27. The USA was Arab’s most main cooperative partner (46, 20.4 %), followed by India (36, 15.9 %).

**Conclusions:**

The amount of literature related to dengue research has considerably increased over the last decade. This bibliometric analysis has demonstrated the leading role that the USA, India, Brazil, Thailand, the UK, and France play in dengue research. The Arab world produced fewer publications related to dengue with lower quality than other world countries.

## Background

Dengue is an important emerging and re-emerging arboviral infection globally as a rapidly growing and widespread public health problem, with transmission occurring in more than 128 countries in Asia, Americas, southeast Africa, western Pacific, and eastern Mediterranean regions [1, 2]. It is estimated that each year, around 390 million people are infected with dengue, of which 96 million develop any level of disease severity [3], leading to approximately 21,000 deaths [3, 4]. The clinical spectrum of dengue infection can vary from asymptomatic to severe manifestations of haemorrhagic fever, hypovolaemic shock, and organ impairment [1, 5]. However, some cases are often accompanied with symptoms such as sore throat, arthralgia, febrile illness anorexia, headaches, myalgia, and a macular skin rash [6–8].

Dengue outbreaks worldwide have progressively increased [9–14], with recent outbreaks in the North Africa and Middle East regions [1, 15, 16]. In recent years, bibliometric analysis has been widely conducted to evaluate scientific research activities in many fields of infectious diseases such as chikungunya [17], Ebola virus disease [18, 19], influenza [20], John Cunningham virus [21], leishmaniasis [22, 23], Mayaro virus fever [24], Malaria [25], yellow fever disease [26], and Zika virus [27]. Bibliometrics is a statistical analysis of written publications and is used to provide quantitative and qualitative analysis of available data deposited at major multidisciplinary journal-indexing database such as Scopus.

Many research fields use bibliometric analysis to assess the scientific research patterns of publication year, document type, countries, journal, impact factors (IF), institution, number of citations, *h*-index, and international collaboration in global trends studies of specific fields [28–34]. It is thought that health research productivity from Arab region is still lagging far behind compared to worldwide publishing production [35–37]. Although the research output in some world regions such as India, China, and Brazil has been evaluated for dengue research [38–40], no study has focused on the scientific output of dengue research in Arab countries relative to that worldwide. Therefore, the aim of this study was to characterize and quantify the scientific output of dengue research in Arab countries relative to that worldwide by using a bibliometric analysis.

## Methods

### Study design

This study used a bibliometric analysis based in previous studies [28, 30, 32–34, 41].

### Data source

This bibliometric study was built on April 2, 2016 based on the online version of Scopus database, which was developed by Elsevier. Note that there are several other databases that could be used in the bibliometric analysis including Web of Science, Google Scholar, and PubMed. However, this study only focuses on the data of Scopus which are assumed to be of the largest database [42, 43].

### Search strategy

For bibliometric analysis, the standardized search approach based on the use of the the keyword “dengue” in the title, abstract, and keyword field was used to get research output related to dengue at a global level. All data related to dengue were collected from the past to December 31, 2015. For further analysis, data retrieved from Scopus were limited to Arab countries, including 22 countries (The Kingdom of Saudi Arabia (KSA), Tunisia, Egypt, Morocco, Algeria, Oman, Iraq, Jordan, United Arab Emirates, Syrian Arab Republic, Lebanon, Qatar, Palestine, Libyan Arab Jamahiriya, Kuwait, Bahrain, Sudan, Yemen, Mauritania, Somalia, Djibouti, and Comoros). The analysed bibliometric indicators included publication year, document type, language of publications, publication distribution by countries/territories, journal, impact factors (IF), institution, number of citations, *h*-index, and international collaboration. As the 2016 data did not represent a complete year, such data were excluded from the analysis. Furthermore, items coded as errata were excluded from further analysis.

### Ethical issues

As there were no humans involved in this study, research using existing data from secondary sources is considered it to be exempt from the institutional review board (IRB) approval process.

### Data analysis

Data downloaded from Scopus were and organized into Microsoft Excel 2007 and then be used for further analysis to get the ten top-ranked for most bibliometric indicators and they were appeared in descending order from 1 to 10 using the standard competition ranking (SCR); (1–2–2–4 rule). Descriptive statistics were used to determine the frequency, percentage, sum, and average. The *h*-index and IF had been used to assess the quality and quantity of research output. The *h*-index was introduced in bibliometric analysis by Hirsch [44] to illustrate the scientific output of a country, organization, researcher, etc. Therefore, the *h*-index covers both the quantity (number of publications) and the impact (number of citations) [45–47]. The impact factor for journals was obtained from the Journal Citation Reports (JCR) © Ranking: 2014 [48].

## Results

**Fig. 1:**
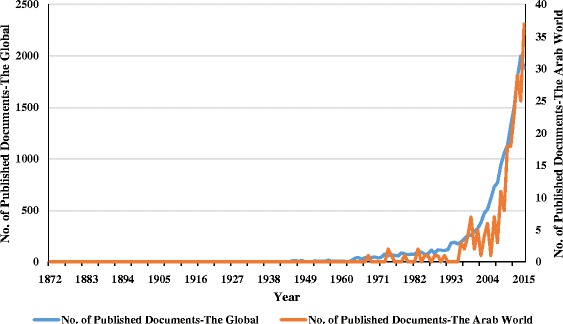
Numbers of dengue research literature trends in Scopus between 1872 and 2015 at the global and Arab levels

There were a total of 19,581 dengue-related documents identified in the Scopus database that were published between 1872 and 2015. These documents were published from the across 12 document types. There were 14,434 paper articles comprising 73.71 % of the total production, followed by reviews (10.26 %), and letters (4.31 %). Other document types such as editorial materials, proceedings papers, notes, and book reviews covered approximately 11.73 % of the published literature. The total publications per year are demonstrated in Fig. 1. The results at global level show that the study of dengue exhibits an overall upward trend from 1872 to 2015 with peak publications in 2014 (data in 2015 may be incomplete as the databases lag, thus, the number of research output in 2015 may be increasing). In the included years, the number of Scopus publications increased very slowly until 1990, and then the numbers of publications noticeably increased after 2000. The publications identified were published in 34 languages. The most commonly used language were English (90.33 %), Spanish (3.57 %), and French (2.59 %).

Table 1 illustrates the top 10 countries in terms of absolute research production, their *h*-index and number of publication from international collaboration. The leading countries in dengue research were the USA (4,709; 24.05 %), India (1,942; 9.92 %), Brazil (1,530; 7.81 %), Thailand (1,260; 6.43 %), the UK (1,129; 5.77 %), and France (1,087; 5.55 %). As expected, most publications related to dengue were carried out in North America, Europe, South Asia, and Latin America. This bibliometric analysis has demonstrated the leading role that the USA, India, Brazil, Thailand, the UK, and France play in dengue research (Table 1).

**Table 1:** Top 10 most prolific countries of publications related to dengue in the world during 1872–2015 (*n* = 19,581)

SCR	Countries	Articles (%)	*h-*index	Collaborations with foreign countries	Number (%)^a^ of publications with international authors
1^st^	United States	4,709 (24.05)	159	146	2,030 (43.11)
2^nd^	India	1,942 (9.92)	52	106	1,012 (52.11)
3^rd^	Brazil	1,530 (7.81)	60	113	440 (28.76)
4^th^	Thailand	1,260 (6.43)	78	69	662 (52.54)
5^th^	United Kingdom	1,129 (5.77)	92	126	803 (71.12)
6^th^	France	1,087 (5.55)	76	147	609 (56.03)
7^th^	Australia	783 (4.00)	68	100	393 (50.19)
8^th^	Singapore	774 (3.95)	59	104	372 (48.06)
9^th^	Malaysia	628 (3.21)	39	108	209 (33.28)
10^h^	Japan	618 (3.16)	48	53	289 (46.76)

*SCR* Standard competition ranking

^a^Percentage of publications with international authors from the total number of publications for each country

In case of number of publications with multinational researchers, France tops the list with 147 countries, followed by the USA with 146 countries, and the UK with 126 countries. Furthermore, the UK (71.12 %) had the highest percentage of documents in collaboration, followed by 56.03 % for France, 52.54 % for Thailand, and 52.11 % for India from the total number of documents for each country. The total number of citations for all publications was 352,710, with an average of 18.0 citations per publication. Furthermore, the *h*-index for all extracted data related to dengue research was 186. The best *h*-index value is achieved by the USA (159) followed by the UK (92), Thailand (78), and France (76); (Table 1).

**Fig. 2:**
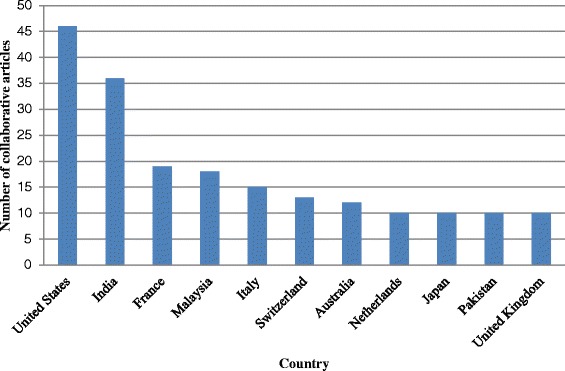
The most 11 internationally collaborative countries/territories with Arab world

The Arab world produced fewer publications related to dengue. Only 226 (1.16 % of the overall global research effort in the dengue field) articles were published from the Arab region across 7 document types. There were 187 paper articles comprising 82.7 % of the total production, followed by reviews (7.5 %), and letters (3.1 %). Editorial materials, proceedings papers, notes, and book reviews covered approximately 6.7 % of the published literature. The total publications per year are demonstrated in Fig. 1. The results from Arab world show that the study of dengue exhibits an overall upward trend from 1968 to 2015 with peak publications in 2015. Table 2 shows that KSA was the most productive country with 102 documents representing 45.1 %, followed by Egypt (58; 25.7 %), Sudan (21; 9.3 %), and Kuwait (21; 9.3 %). No published data related to dengue were available from Comoros, and Mauritania. In Arab world, KSA and Egypt were at positions 34 and 46 respectively. The Arab countries have cooperated with 87 countries/ territories in the field of dengue research. The most internationally collaborative countries/ territories appears in Fig. 2. The total number of citations that got for publications (*n* = 133) from collaboration was 1,918. The USA was Arab’s most main cooperative partner (46, 20.4 %), followed by India (36, 15.9 %), France (19, 8.4 %) and Malaysia (18, 8 %). The total number of citations for all publications was 2,683, with an average of 12 citations per publication. Furthermore, the *h*-index for all extracted data related to dengue research was 27. In addition, Egypt achieved the highest *h*-index (value of *h*-index =15).

**Table 2:** Bibliometric analysis of the 226 documents from the Arab world during 1968–2015

SCR^a^	Countries	Total number of articles for the whole period (%)	*h-index*	Number of documents with international collaboration
1^st^	KSA	102 (45.1)	11	68
2^nd^	Egypt	58 (25.7)	15	49
3^rd^	Sudan	21 (9.3)	8	11
3^rd^	Kuwait	21 (9.3)	11	14
5^th^	Tunisia	14 (6.2)	7	11
6^th^	UAE	10 (4.4)	6	8
6^th^	Oman	10 (4.4)	3	5
8^th^	Yemen	7 (3.1)	5	7
8^th^	Morocco	7 (3.1)	5	3
10^th^	Qatar	6 (2.7)	2	6
11^th^	Jordan	5 (2.2)	5	4
11^th^	Bahrain	5 (2.2)	2	4
13^th^	Algeria	4 (1.8)	2	3
13^th^	Lebanon	4 (1.8)	3	3
15^th^	Palestine	3 (1.3)	3	3
16^th^	Iraq	2 (0.9)	2	2
16^th^	Somalia	2 (0.9)	2	2
16^th^	SAR	2 (0.9)	2	2
16^th^	Djibouti	2 (0.9)	1	1
20^th^	LAJ	1 (0.4)	1	1

*SCR* Standard competition ranking, *KSA* Kingdom of Saudi Arabia, *UAE* United Arab Emirates, *SAR* Syrian Arab Republic, *LAJ* Libyan Arab Jamahiriya

^a^Equal countries have the same ranking number, and then a gap is left in the ranking numbers

The top 10 most productive journals based on the number of publication at global level were analyzed (Table 3). The 10 most prolific journals together produced 3,535 publications, comprising 18.05 % of worldwide researchers’ contributions during 1872–2015. The *American Journal of Tropical Medicine and Hygiene* published the most publications (755, 3.86 %) by worldwide researchers, followed by the *Plos Neglected Tropical Diseases*, with 494 (2.52 %), *Southeast Asian Journal of Tropical Medicine and Public Health* with 393 (2.01), and *Plos One* with 391 (2.00 %). Most journals in the list of the top ranking journals (9 journals out of 10) had IF. The IF for journals ranged from 0.719–6.751. Furthermore, the top 10 most productive journals based on the number of publication at Arab world level were analyzed (Table 4). The 10 most prolific journals together produced 67 papers, comprising 29.6 % of Arab authors’ contributions during 1968–2015. The *Parasitology Research*, and *Dengue Bulletin* published the most publications (9 publications for each journal, (4.0 %)) by Arab authors, followed by the *Eastern Mediterranean Health Journal*, with 7 (3.1 %). Most journals in the list of the top ranking journals (7 journals out of 11) had IF. The IF for journals ranged from 1.839 to 3.247.

**Table 3:** The 10 most published journals worldwide during 1968–2015 (*n* = 19,581)

SCR	Journal	Frequency (%)	IF^a^
1^st^	*American Journal of Tropical Medicine and Hygiene*	755 (3.86)	2.699
1^st^	*Plos Neglected Tropical Diseases*	494 (2.52)	4.446
3^rd^	*Southeast Asian Journal of Tropical Medicine and Public Health*	393 (2.01)	0.719
4^th^	*Plos One*	391 (2.00)	3.234
5^th^	*Journal of Virology*	380 (1.94)	4.439
6^th^	*Dengue Bulletin*	254 (1.30)	NA
7^th^	*Virology*	229 (1.17)	3.321
8^th^	*Emerging Infectious Diseases*	223 (1.14)	6.751
9^th^	*Transactions of the Royal Society of Tropical Medicine and Hygiene*	212 (1.08)	1.839
10^th^	*Vaccine*	204 (1.04)	3.624

*SCR* Standard competition ranking, *NA* Not available, *IF* Impact factor

^a^The impact factor was reported according to the journal citation reports (JCR) 2014

**Table 4:** The 10 most published journals from the Arab world during 1968–2015 (*n* = 226)

SCR^a^	Journal	Frequency (%)	IF^b^
1^st^	*Parasitology Research*	9 (4.0)	2.098
1^st^	*Dengue Bulletin*	9 (4.0)	NA
3^rd^	*Eastern Mediterranean Health Journal*	7 (3.1)	NA
4^th^	*Journal of Infection and Public Health*	6 (2.7)	NA
4^th^	*Biosciences Biotechnology Research Asia*	6 (2.7)	NA
4^th^	*American Journal of Tropical Medicine and Hygiene*	6 (2.7)	2.699
4^th^	*Transactions of the Royal Society of Tropical Medicine and Hygiene*	6 (2.7)	1.839
8^th^	*Journal of Medical Virology*	5 (2.2)	2.347
8^th^	*BMC Infectious Diseases*	5 (2.2)	2.613
10^th^	*Plos One*	4 (1.8)	3.234
10^th^	*FEMS Immunology and Medical Microbiology*	4 (1.8)	3.078

*SCR* Standard competition ranking, *NA* Not available, *IF* Impact factor

^a^Equal journals have the same ranking number, and then a gap is left in the ranking numbers

^b^The impact factor was reported according to the journal citation reports (JCR) 2014

At global level, the number of citations ranged from 1451 for the top cited article to 645 for the 10^th^. The most frequently cited article was published in 1998 in *Clinical Microbiology Reviews* (IF = 17.406) by Gubler [49], and cited 1451 times in Scopus database. The other 9 publications that were the most frequently cited in the field of dengue research at global level are shown in Table 5 [3, 49–57]. At Arab level, the number of citations ranged from 162 for the top cited article to 59 for the 10^th^. The most frequently cited article was published in 2006 in *Transactions of the Royal Society of Tropical Medicine and Hygiene* (IF = 1.839, 2014) by Boutayeb [58], and cited 162 times in Scopus database. The other 9 publications that were the most frequently cited in the field of dengue research at Arab level are shown in Table 6 [59–67].

**Table 5:** Top 10 most cited articles in *Scopus* related to dengue worldwide

SCR	Authors	Title	Year of publication	Source title	Cited by
1^st^	Gubler [49]	Dengue and dengue hemorrhagic fever	1998	*Clinical Microbiology Reviews*	1451
2^nd^	Bhatt et al. [3]	The global distribution and burden of dengue	2013	*Nature*	991
3^rd^	Halstead [50]	Pathogenesis of dengue: Challenges to molecular biology	1988	*Science*	991
4^th^	Lanciotti et al. [51]	Rapid detection and typing of dengue viruses from clinical samples by using reverse transcriptase-polymerase chain reaction	1992	*Journal of Clinical Microbiology*	896
5^th^	Vaughn et al. [52]	Dengue viremia titer, antibody response pattern, and virus serotype correlate with disease severity	2000	*Journal of Infectious Diseases*	807
6^th^	Gubler [53]	Epidemic dengue/dengue hemorrhagic fever as a public health, social and economic problem in the 21st century	2002	*Trends in Microbiology*	749
7^th^	Halstead [54]	Dengue	2007	*Lancet*	720
8^th^	Guzmán and Kourí [55]	Dengue: An update	2002	*Lancet Infectious Diseases*	718
9^th^	Kuhn et al. [56]	Structure of dengue virus: Implications for flavivirus organization, maturation, and fusion	2002	*Cell*	682
10^th^	Mackenzie et al. [57]	Emerging flaviviruses: The spread and resurgence of Japanese encephalitis, West Nile and dengue viruses	2004	*Nature Medicine*	645

*SCR* Standard competition ranking

**Table 6:** Top 10 most cited articles in *Scopus* related to dengue from Arab world

SCR^a^	Authors	Title	Year of publication	Source title	Cited by
1^st^	Boutayeb [58]	The double burden of communicable and non-communicable diseases in developing countries	2006	*Transactions of the Royal Society of Tropical Medicine and Hygiene*	162
2^nd^	Chaturvedi et al. [59]	Cytokine cascade in dengue hemorrhagic fever: Implications for pathogenesis	2000	*FEMS Immunology and Medical Microbiology*	156
3^rd^	Raghupathy et al. [60]	Elevated levels of IL–8 in dengue hemorrhagic fever	1998	*Journal of Medical Virology*	108
4^th^	Graham et al. [61]	A prospective seroepidemiologic study on dengue in children four to nine years of age in Yogyakarta, Indonesia I. Studies in 1995–1996	1999	*American Journal of Tropical Medicine and Hygiene*	104
5^th^	Agarwal et al. [62]	A clinical study of the patients with dengue hemorrhagic fever during the epidemic of 1996 at Lucknow, India	1999	*Southeast Asian Journal of Tropical Medicine and Public Health*	80
6^th^	Moutailler et al. [63]	Potential vectors of rift valley fever virus in the Mediterranean region	2008	*Vector-Borne and Zoonotic Diseases*	70
7^th^	Chaturvedi et al. [64]	Sequential production of cytokines by dengue virus-infected human peripheral blood leukocyte cultures	1999	*Journal of Medical Virology*	68
7^th^	Mustafa et al. [66]	Elevated levels of interleukin–13 and IL–18 in patients with dengue hemorrhagic fever	2001	*FEMS Immunology and Medical Microbiology*	68
9^th^	Abubakar et al. [65]	Global perspectives for prevention of infectious diseases associated with mass gatherings	2012	*The Lancet Infectious Diseases*	63
10^th^	Barniol et al. [67]	Usefulness and applicability of the revised dengue case classification by disease: Multi-centre study in 18 countries	2011	*BMC Infectious Diseases*	59

*SCR* Standard competition ranking

^a^Equal articles have the same ranking number, and then a gap is left in the ranking numbers

Table 7 provides a list of the top 10 most productive institutes at global level between 1872 and 2015. *Mahidol University* in Thailand ranked first in the number of publications (620, 3.17 %), followed by *Fundacao Oswaldo Cruz* in Brazil (495, 2.53 %), and *Centers for Disease Control and Prevention* in the USA (395, 2.02 %). At Arab level, the top 10 most productive institutions for total publications are shown in Table 8. *King Abdulaziz University* in KSA ranked first in the number of publications (32, 14.2 %), followed by *U.S. Naval Medical Research Unit No. 3* in Egypt (17, 7.5 %), *Ministry of Health* in KSA (17, 7.5 %), and Faculty of Medicine in Kuwait (17, 7.5 %).

**Table 7:** The top 10 most productive institutes at global level

SCR	Institution, country	No. of documents (%)
1^st^	*Mahidol University*, Thailand	620 (3.17)
2^nd^	*Fundacao Oswaldo Cruz*, Brazil	495 (2.53)
3^rd^	*Centers for Disease Control and Prevention*, USA	395 (2.02)
4^th^	*Instituto de Medicina Tropical Pedro Kouri*, Cuba	309 (1.58)
5^th^	*University of Malaya*, Malaysia	297 (1.52)
6^th^	*Institut Pasteur, Paris*, France	294 (1.50)
7^th^	*Universidade de Sao Paulo – USP,* Brazil	274 (1.40)
8^th^	*Walter Reed Army Institute of Research,* USA	255 (1.30)
9^th^	*Armed Forces Research Institute of Medical Sciences,* Thailand	250 (1.28)
10^th^	*University of Oxford,* UK	230 (1.17)

*SCR* Standard competition ranking

**Table 8:** The top 10 most productive institutes from or collaborating with Arab world affiliations during the study period

SCR^a^	Institution, country	No. of documents (%)
1^st^	*King Abdulaziz University*, KSA	32 (14.2)
2^nd^	*U.S. Naval Medical Research Unit No. 3 (NAMRU–3),* Egypt	17 (7.5)
2^nd^	*Ministry of Health*, KSA	17 (7.5)
2^nd^	*Faculty of Medicine*, Kuwait	17 (7.5)
5^th^	*Organisation Mondiale de la Sante*, Switzerland	14 (6.2)
6^th^	*Umm Al Qura University*, KSA	10 (4.4)
6^th^	*King George’s Medical University*, India	10 (4.4)
6^th^	*Universita di Pisa*, Italy	10 (4.4)
9^th^	*King Saud University*, KSA	9 (4.0)
10^th^	*Bharathiar University*, India	8 (3.5)
10^th^	*Universita degli Studi di Roma La Sapienza*, Italy	8 (3.5)

*SCR* Standard competition ranking, *KSA* Kingdom of Saudi Arabia

^a^Equal institutes have the same ranking number, and then a gap is left in the ranking numbers

## Discussion

In this study, I employed a bibliometric approach to analyse the research trends of dengue at global level and in the Arab world. The publications on dengue presented a solid growth with an increasing number of articles. This bibliometric analysis has demonstrated the leading role that the USA, India, Brazil, Thailand, the UK, and France play in dengue research. USA was the leading country in research output on dengue. Although the number of dengue outbreaks in the USA there is less than in other countries [3], these findings are in line with those of previous studies, especially those in other infectious fields [17, 18, 23, 24]. Overall, Brazil, India, Thailand, Singapore, Malaysia, Japan, and Australia, accounted for most of the dengue research activity at global level. A possible explanation for these results may be due to high prevalence of dengue in these countries which faced many outbreaks [68–74]. Another possible explanation for these findings could be recognized to the number of researchers and development of scientific research system in these countries. However, only KSA and Egypt among Arab countries ranked among the first 50 countries in terms of worldwide contribution to research productivity in the dengue. Among the Arab countries, KSA and Egypt achieved the top rank. High national incomes and large populations and are the most probable reasons for this achievement. These results are consistent with data obtained from previous studies, especially those in medical fields [29, 31, 37, 75–81]. Publications from Arab world received lower citation rate than that from the world; because researchers from Arab world have published their work in scientific journals with slightly lower citation rates or without IF such as *Dengue Bulletin*, *Eastern Mediterranean Health Journal*, *Journal of Infection and Public Health; and Biosciences Biotechnology Research Asia.* These findings are in line with those of previous studies [75, 77, 78, 82].

The current study designed to recognize scientific collaborations between Arab countries and non-Arab countries. The USA was Arab’s most main cooperative partner. These results match those observed in earlier studies in the field of ophthalmology [77], and in the field of substance use disorders [83]. In addition, country with highest collaboration with researchers in India in dengue research was USA [84]. The investigation of publication output recognized several successful cases of researcher collaboration between Arab countries and Western Europe (France and Italy) and the Asiatic region (i.e. India and Malaysia). At the global level, the USA got a leadership position in dengue research with the largest publication followed by India [85]. Dengue disease has no borders and prevention; eradication and control of this disease requires worldwide efforts. Health-care systems in the Arab region are low priority in national spending plans and are perceived as being non-productive [86, 87]. Arab region has been the source of most fatal infectious diseases (e.g. Middle East respiratory syndrome coronavirus, West Nile Virus, and leishmaniasis), and they should heavily participate and cooperate in research in order to combat them [75]. The Arab countries have cooperated with 87 countries in the field of dengue research.

The current study is the first to assess the quantity and quality of global research effort in the dengue field worldwide and from Arab world. The most important limitation lies in the fact that the Scopus database only was used to extract data related to dengue. Articles published in non Scopus-cited journals were not studied, but it is interesting to note that the Scopus has several advantages more than others, as it is the largest abstract and citation database of peer-reviewed literature [42, 43, 88].

## Conclusions

Based on 19,581 dengue-related documents from Scopus, this bibliometric analysis provided an overview of research in dengue and recognized some noteworthy issues in this field during the search period. The amount of literature related to dengue research has considerably increased over the last decade. This bibliometric analysis has demonstrated the leading role that the USA, India, Brazil, Thailand, the UK, and France play in dengue research. The Arab world produced fewer publications related to dengue with lower quality than other world countries. The result shows that KSA plays a leading role in dengue research through the number of publications with international collaboration in Arab world. The USA was Arab’s leading internationally collaborative country, followed by India, France and Malaysia. Multinational collaboration can help dengue research get more international attention. More research is required to recognize what societal and individual level factors were involved in raising such a remarkable increase in quantity of dengue research in the last decade. Arab researchers especially in countries who at risk with dengue disease need to take the lead and promote research projects in this field of infection as an important public health concern.

## Abbreviations

IF: impact factor
IRB: institutional review board
JCR: Journal citation reports
KSA: Kingdom of Saudi Arabia
SCR: Standard competition ranking
